# Number is not just an illusion: Discrete numerosity is encoded independently from perceived size

**DOI:** 10.3758/s13423-021-01979-w

**Published:** 2021-08-11

**Authors:** Andrea Adriano, Luisa Girelli, Luca Rinaldi

**Affiliations:** 1 Department of Psychology, University of Milano-Bicocca, Milano, Italy; 2 NeuroMI, Milan Center for Neuroscience, Milano, Italy; 3 Department of Brain and Behavioral Sciences, University of Pavia, Pavia, Italy; 4 Cognitive Psychology Unit, IRCCS Mondino Foundation, Pavia, Italy; 5 Dipartimento di Psicologia, Università degli Studi di Milano-Bicocca, Piazza dell’Ateneo Nuovo 1, Edificio U6, 20126 Milano, Italy

**Keywords:** Ponzo illusion, Kanizsa illusion, Approximate number system, Numerosity processing

## Abstract

While seminal theories suggest that nonsymbolic visual numerosity is mainly extracted from segmented items, more recent views advocate that numerosity cannot be processed independently of nonnumeric continuous features confounded with the numerical set (i.e., such as the density, the convex hull, etc.). To disentangle these accounts, here we employed two different visual illusions presented in isolation or in a merged condition (e.g., combining the effects of the two illusions). In particular, in a number comparison task, we concurrently manipulated both the perceived object segmentation by connecting items with Kanizsa-like illusory lines, and the perceived convex-hull/density of the set by embedding the stimuli in a Ponzo illusion context, keeping constant other low-level features. In Experiment 1, the two illusions were manipulated in a compatible direction (i.e., both triggering numerical underestimation), whereas in Experiment 2 they were manipulated in an incompatible direction (i.e., with the Ponzo illusion triggering numerical overestimation and the Kanizsa illusion numerical underestimation). Results from psychometric functions showed that, in the merged condition, the biases of each illusion summated (i.e., largest underestimation as compared with the conditions in which illusions were presented in isolation) in Experiment 1, while they averaged and competed against each other in Experiment 2. These findings suggest that discrete nonsymbolic numerosity can be extracted independently from continuous magnitudes. They also point to the need of more comprehensive theoretical views accounting for the operations by which both discrete elements and continuous variables are computed and integrated by the visual system.

**Supplementary Information:**

The online version contains supplementary material available at 10.3758/s13423-021-01979-w.

Main computational, psychophysical, and neuroimaging studies (e.g., Burr & Ross, 2008; Dehaene & Changeux, 1993; Piazza et al., 2004; Stoianov & Zorzi, 2012; Verguts & Fias, 2004) have maintained the existence of a dedicated approximate number system (ANS) that, in the visual domain, would directly extract numerosity from the retinal input through a primitive visual segmentation and individuation algorithm (Dehaene & Changeux, 1993). According to this view, also known as the number sense theory, human adults are endowed with an innate cognitive mechanism shared with children (e.g., Brannon et al., 2004; Xu & Spelke, 2000) and animals (e.g., Agrillo et al., 2012; Brannon & Terrace, 1998; Nieder & Miller, 2004), allowing nonsymbolic numerosity to be processed independently of continuous magnitudes confounded with numerosity (e.g., convex hull, density, area) following the psychophysical Weber’s law (Whalen et al., 1999).

However, an ongoing and fervent theoretical debate has emerged in the past years regarding which exact visual features are employed by the ANS to extract an approximate analog representation of number. Indeed, grounding on the evidence that performance in numerical tasks is affected by the manipulation of several physical continuous features confounded with numerosity (e.g., Allik & Tuulmets, 1991; Chakravarthi & Bertamini, 2020; Dakin et al., 2011; Durgin, 2008; Gebuis & Reynvoet, 2012a, 2012b; Hurewitz et al., 2006; Katzin et al., 2020), more recent views (i.e., the so-called indirect accounts) have suggested that people would not extract numerical information independently from these continuous magnitudes (e.g., Gebuis & Reynvoet, 2012a, 2012b).

Disentangling the contribution of discrete information (e.g., the number of segmented entities in the set or numerosity) from continuous visual features confounded with numerosity (e.g., convex hull, density, area) therefore represents the main theoretical and experimental challenge to probe which visual mechanisms and sensory features are exploited by the ANS to reach an approximate numerical representation (Gebuis et al., 2016; Leibovich et al., 2017). Visual illusions could be the ideal tool to dissociate the subjective perception of (discrete) numerosity from continuous features because they help to reveal the relationship between physical stimulation (e.g., at the retinal level) and the subjective perception of the visual input. Therefore, they can be used to selectively manipulate a visual feature without compromising other physical visual features in the image (e.g., Picon et al., 2019). For instance, the connectedness illusion has been used to manipulate the level of perceived segmentation of the items in a set, keeping constant the low-level features across connectedness levels (Adriano, Girelli, et al., 2021; Adriano, Rinaldi, et al., 2021; Franconeri et al., 2009; Kirjakovski & Matsumoto, 2016). In particular, some of these studies employed Kanizsa-like illusory contour lines (e.g., Nieder, 2002) to connect the dots in the set. Results showed that increasing the illusory connected dot pairs proportionally reduced the perceived numerosity (i.e., as a function of the number of illusory connections). This is likely to emerge because the visual system processes two connected dots as a single unified perceptual object (e.g., Anobile et al., 2017; Franconeri et al., 2009), as maintained by the grouping principle of element connectedness (Palmer & Rock, 1994). These findings thus suggest that nonsymbolic numerosity would be extracted from discrete, segmented (perceptual) objects rather than from raw, low-level features of an unsegmented scene.

By contrast, other studies manipulating the perceived size of continuous features by means of size illusions bring evidence in favor of the indirect account (Dormal et al., 2018; Picon et al., 2019). Size illusions are perceptual phenomena in which the physical size of a stimulus is altered by contextual cues. For instance, Picon et al. (2019) contingently manipulated numerosity and perceived size, embedding numerical arrays in the classic Ebbinghaus illusion context. Results showed that participants significantly overestimated the number of dots presented in a *perceived* larger convex hull and underestimated the number of dots presented in the *perceived* smaller convex hull. Accordingly, and in line with indirect accounts, they suggested that numerosity would be mainly encoded through continuous physical features (e.g., convex hull/density).

Despite these previous studies employing different visual illusions seeming to reach contradictory conclusions, it is worth noting that they used only one type of illusion at time, targeting, in turn, different key visual information in the stimuli. That is, studying the effect of visual illusions *in isolation* does not provide much insight regarding whether (i) one type of information (i.e., discrete elements or continuous variables) prevails over the other or (ii) both types of information independently contribute to numerosity perception. To this aim, in the present study, we *concurrently* applied two different visual illusions over the same stimuli to shed light on the processing of visual discrete numerosity information and continuous physical features. A similar approach, combining visual illusions in the same stimulus, has been already employed to investigate the extent to which different simultaneous visual distortions may interact affecting the final percept, a condition not uncommon in real-world perception and in visual arts such as drawing (e.g., Ni, 1934; Coren & Ward, 1979). In particular, here we employed the Kanizsa illusion to manipulate the perceived item segmentation as well as the Ponzo illusion, a geometrical optical illusion, to manipulate the perceived convex hull/density of the set. We independently modulated the direction of each illusion bias (e.g., underestimation or overestimation) but, crucially, keeping constant at the same time all the physical and contextual cues across key experimental conditions. Hence, illusions were presented in isolation or in a merged condition (e.g., combining the effects of the two illusions).

## Experiment 1

In the Experiment 1, participants performed a number comparison task in which we manipulated the effect of the two illusions, obtaining four different experimental conditions: one condition without illusions (e.g., baseline), one condition with only the Kanizsa illusion, one condition with only the Ponzo illusion, and one combined (or merged) condition, with illusions triggering a bias in the same direction (e.g., both acting toward an underestimation bias). If numerosity is processed independently from continuous magnitudes, we should find the larger underestimation in the combined condition compared with the single illusion conditions. On the contrary, according to the indirect account, the bias in the combined condition should not differ from the bias in the condition with only the Ponzo illusion, as the perceived convex hull/density should play the leading role in driving numerosity estimation.

### Materials and methods

#### Participants

Due to COVID-19 restrictions in Italy, the participants were recruited through Pavlovia (www.pavlovia.org), a repository and launch platform allowing online experiments. A total sample of 67 participants (*M*_age_ = 33.8 years, *SD* = 11.4 years, 46 females, 57 right-handed) took part in the study. All participants had normal or correct-to-normal vision and were naïve about the purpose of the experiment. The study was approved by the Local Ethical Committee (protocol N° RM-2020-230).

#### Stimuli and design

The experimental stimuli were generated off-line by a custom Python/PsychoPy script (Peirce, 2007) and were constructed with the same specifications as in Adriano et al. (2021), adding the specific context lines forming the Ponzo illusion.

The whole experimental set was composed of 168 test patterns (42 random spatial patterns cloned across four illusion conditions) and of 168 reference patterns (42 random spatial patterns repeated four times to match the four illusion conditions). The reference patterns always contained the same numerosity (*N* = 12), consisting of 12 black “Pac-Man”-like items (diameter = 20 pixels; notch width = 4 pixels; notch length = 10 pixels, measured from the center; RGB = −1, −1, −1) spatially scattered and randomly rotated at an angle varying across 360° to avoid collinearities and pop-out of illusory contours (ICs). The test patterns contained a variable numerosity, that is, from nine to 15 “Pac-Man”-like items. Half of the test patterns (*N* = 84) were composed by “Pac-Man”-like items that were not eliciting any ICs, while in the other half of test patterns (*N* = 84) “Pac-Man”-like items were purposely aligned to prompt ICs (i.e., the Kanizsa illusion).

**Fig. 1:**
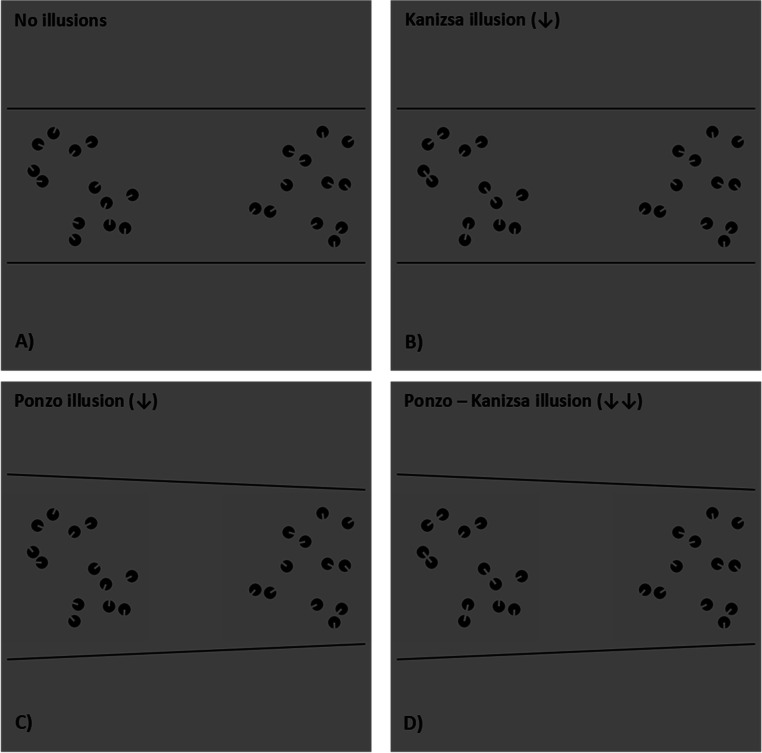
The four experimental conditions of Experiment 1. **a** The no-illusions condition, in which neither the Kanizsa nor the Ponzo illusions were presented. **b** The Kanizsa illusion condition, in which only the effect of items connectedness was manipulated. **c** The Ponzo illusion condition, in which the perceived convex-hull/density of the sets was manipulated. **d** The Ponzo–Kanizsa merged illusion, combining the effects of these last two conditions. In the example reported, the test set (nine to 15 items) is positioned on the left side of the screen (i.e., and hence, in the larger side of the Ponzo illusion), while the reference set (always 12 items) is presented on the right side (i.e., and hence, in the smaller side of the Ponzo illusion). The small arrows represent the direction of the predicted bias of each illusion: in particular, an underestimation is expected for both the Kanizsa and the Ponzo illusions; moreover, if the effects of the two illusions (and thus the effects of segmentation mechanisms and continuous variables) would be additive, the greater underestimation should be observed in the merged condition

Overall, four different experimental conditions were designed (see Fig. 1 for a graphical depiction), according to the specific test pattern employed: (a) a no-illusions condition, in which neither the Kanizsa nor the Ponzo illusions were presented (i.e., the “Pac-Man” items did not trigger any ICs, and the sets were embedded in two parallel lines); (b) a Kanizsa illusion condition, in which only the effect of items connectedness was manipulated (i.e., the “Pac-Man” items were aligned to trigger ICs, and the sets were embedded in two parallel lines; in this case, an underestimation is thus expected); (c) a Ponzo illusion condition, in which only the perceived convex-hull/density of the sets was manipulated (i.e., the “Pac-Man” items did not trigger any ICs, but the sets were embedded in two tilted lines; also, here, an underestimation is expected, as the test set was always anchored in the larger part of the Ponzo illusory context); (d) a combined or merged Ponzo–Kanizsa illusion condition, in which both the effects of items connectedness and the perceived convex-hull/density of the sets were manipulated (i.e., the “Pac-Man” items were aligned to trigger ICs, and the sets were embedded in two tilted lines; in this combined condition, a greater underestimation is expected).

In particular, a first set of 42 test patterns was generated for the no-illusions condition (six random visual patterns were generated for each of the seven numerosity values in test stimuli), which were coupled to the 42 reference patterns. In each test pattern of the no-illusions condition set, all the inducers were not aligned (did not trigger ICs). Each of the 42 stimuli pairs of the no-illusions condition (i.e., composed of reference and stimulus patterns) were embedded inside two parallel black lines (width = 2 pixels; RGB = −1, −1, −1) forming a rectangle whose base was 580 pixels and the height 250 pixels placed at the screen center. These two parallel lines did not elicit any illusion (and were used as a control for the Ponzo illusion).

In the Kanizsa illusion condition, to keep constant spatial profiles of test sets from the baseline (i.e., and thus to control continuous variables), each different test pattern for each numerosity of the no-illusions condition was cloned. Thus, we kept constant the spatial position of all the single items in a given test pattern from the no-illusions set. Critically, in this case a subset of “Pac-Man” items was appropriately rotated and aligned to prompt four ICs for the Kanizsa condition. The distance between the “Pac-Man” items that could prompt the required number of ICs for the connectedness (or Kanizsa) condition was randomly chosen among four possible values (center-to-center distance = 22, 25, 28, and 31 pixels). In this way, the 42 different reference patterns were associated with the same spatial pattern of test stimuli across the no-illusions and the Kanizsa illusion condition. In both conditions, test and reference stimuli were embedded inside two parallel black lines, so that no Ponzo illusion was prompted.

Then, these two conditions were cloned and drawn embedded in the Ponzo illusion context, thus generating stimuli pairs for the Ponzo illusion condition and the combined Ponzo–Kanizsa illusion condition. The Ponzo illusion was elicited by two tilted black lines (width = 2 pixels; RGB = −1, −1, −1) forming the legs of an isosceles trapezoid whose virtual longer base was 300 pixels length and whose shorter base was 250 pixels length (distance between the bases of 580 pixels). Note that in the experiment, the relative positions of the reference and the test stimuli were randomized between the left and right side. Yet the test set was always anchored in the larger part of the Ponzo illusion context, which was randomized in accordance with the position of the test stimulus, so that when the test stimulus appeared to the right, the Ponzo illusion context was drawn with the larger side on the right side.

All the patterns in the four experimental conditions were drawn on a grey background (RGB = 0, 0, 0) and reference and test stimuli were projected within two virtual squared panels (240 × 240 pixels) centered at ±156 pixels from the screen center. Furthermore, we constrained the single “Pac-Man” items in test and in reference stimuli to be distant at least 20 pixels from the four virtual square edges and to not overlap with each other (minimum center-to-center distance = 22 pixels).

#### Procedure

The stimuli were presented by means of an online PsychoPy routine (Peirce, 2007), and all the experimental materials (stimuli, etc.) were downloaded and stored on the computer of each participant. The general procedure was explained to each participant before starting the experiment by means of detailed instructions provided on the display. No information about the illusions was given to the participant.

**Fig. 2:**
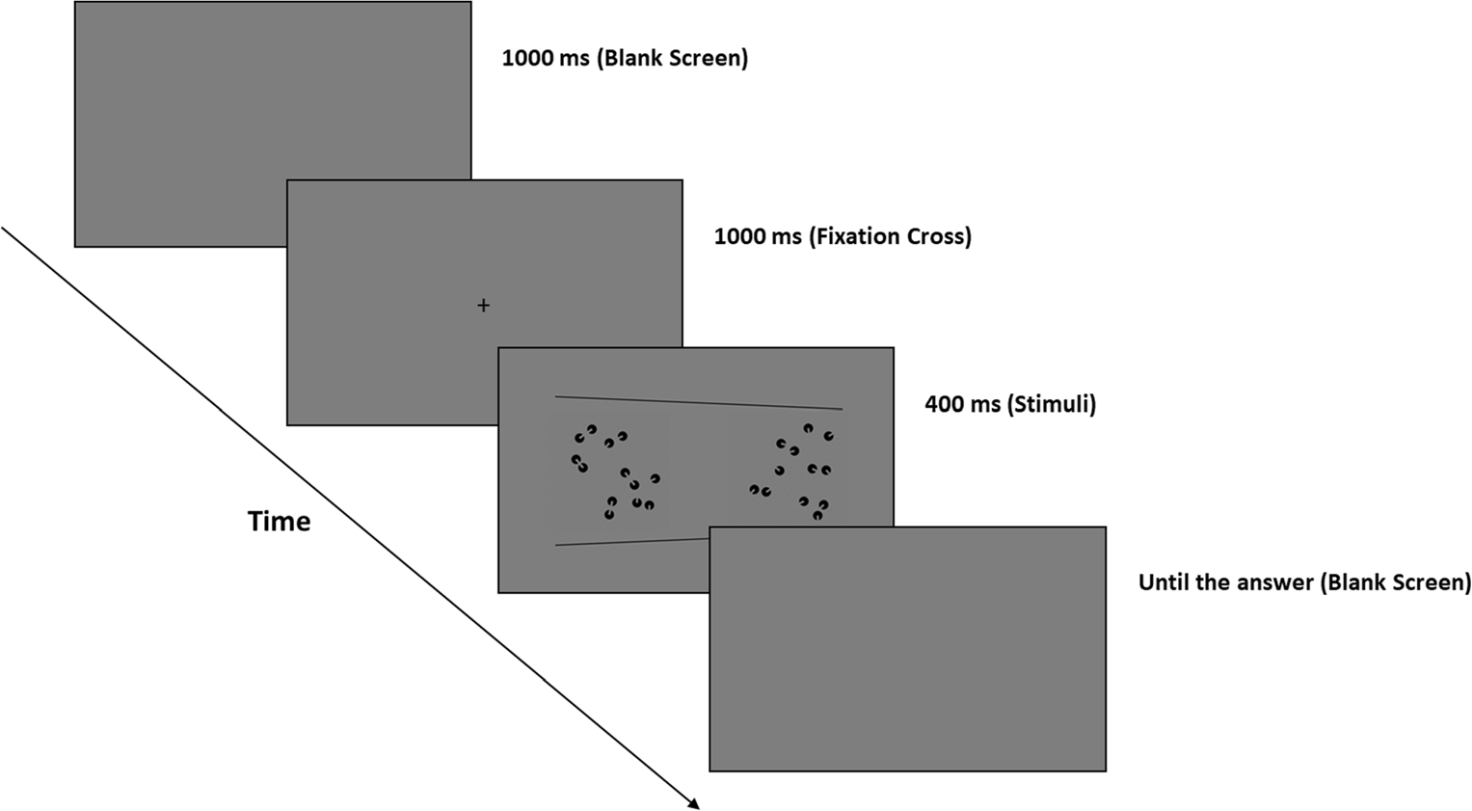
The numerical comparison task. Participants had to indicate the numerically larger between the two collections of dots by pressing the corresponding left or right key

The participants performed a two-alternative forced-choice task, in which they were asked to choose the set containing more dots between two rapidly presented visual patterns by pressing the corresponding keys on the keyboard. The experimental phase was preceded by a brief training composed of 24 trials (six trials for each of the four illusion contexts) to allow the subject to familiarize with the task. In the training phase, we presented only the reference patterns versus the test pattern with nine items. Each experimental trial started with a middle-grey background (RGB = 0, 0, 0) lasting 1,000 ms, followed by a black fixation cross (font: Times; size: 16 pixels; RGB = −1, −1, −1) projected for 1,000 ms, and then, two collections of dots appeared at the left and right of the center of the screen (i.e., the two collections were centered at ±156 pixels from the screen center) for additional 400 ms (see Fig. 2). The side of the reference and test patterns was counterbalanced and randomized across trials. Test set was anchored to the larger side of the Ponzo illusion, which was randomized accordingly to the left or to the right, following the test stimulus side. After the stimuli offset, an empty screen (RGB = 0, 0, 0) was presented until the participant’s answer. The subjects could select the stimulus by pressing the appropriate key with their left or right index finger (“F” key for the left stimulus and “J” key for the right stimulus).

Response time was not restricted, but we emphasized in the instructions to answer as fast as possible. After the practice session, two counterbalanced blocks (i.e., across participants) composed of 168 randomly ordered trials were presented, for a total of 336 experimental trials (12 trials for each of the seven numerosities across the four illusion contexts), separated by a self-paced pause at the half of the whole session. The whole experiment lasted around 15–20 min.

#### Data analysis

The data were analyzed with R-Studio (RStudio Team, 2018, Version 3.6.2; http://www.rstudio.com/) and Jamovi (The Jamovi Project, 2019, Version 1.1.5; https://www.jamovi.org) softwares. Psychometric functions for each condition were generated by fitting Gaussian cumulative distribution functions to the data, and parameters were estimated with a parametric approach based on maximum likelihood method, using Quickpsy package for R (Linares & López-Moliner, 2016). In order to minimize biases in estimating the psychometric function parameters, we fitted the psychometric curves taking into account the typical lapse in performance (e.g., missing a trial, finger errors) by allowing the value of the guess rate (γ) and lapse rate (λ) parameters to vary in the default range of 0–0.05 (Wichmann & Hill, 2001).

To investigate the effect of the illusions over perceived numerosity, we calculated the point of subjective equality (PSE) for each illusion condition as a function of the numerosity in test set—that is, the number of dots in test patterns required in order to be subjectively judjed as equal to the the reference patterns (12 items). The 50% of the chosen test patterns was set as threshold level. The 95% confidence intervals of individual PSEs were estimated running 200 bootstrap resampling of the data. Furthermore, as an index of the precision of the numerical discrimination and to confirm that the performance follows Weber’s law (e.g., JND/N = *k*) we calculated the coeffienct of variation (CoV; Whalen et al., 1999), as the ratio between the standard deviation (*SD*) and the PSE of the psichometric functions for each illusion condition. Reaction times (RTs) for each illusions condition were also recorded. RTs’ data were logarithmically transformed and responses whose latencies fell outside of 1.5 times the interquartile range of the distribution were discarded (a total 4.89 % of the trials were discarded from RTs data). Two separated one-way repeated-measures analyses of variance (ANOVAs) were performed with the experimental condition (no illusion, Kanizsa, Ponzo, Ponzo–Kanizsa) as within-subjects factor and with the mean PSE or the mean CoV as dependent variables. Furthermore, we performed a 4 × 4 repeated-measures ANOVA, with the absolute numerical distance between reference and test stimuli (0, 1, 2, 3) and the experimental condition (no illusion, Kanizsa, Ponzo, Ponzo–Kanizsa) as within-subjects factors and the mean RTs as dependent variable. The Greenhouse–Geisser epsilon (ε) correction for violation of sphericity was applied when needed and original *F*, *df*, and corrected *p* values were reported. Frequentist analyses were also accompanied by respective Bayesian analysis in the case of nonsignificant results.

### Results

**Fig. 3:**
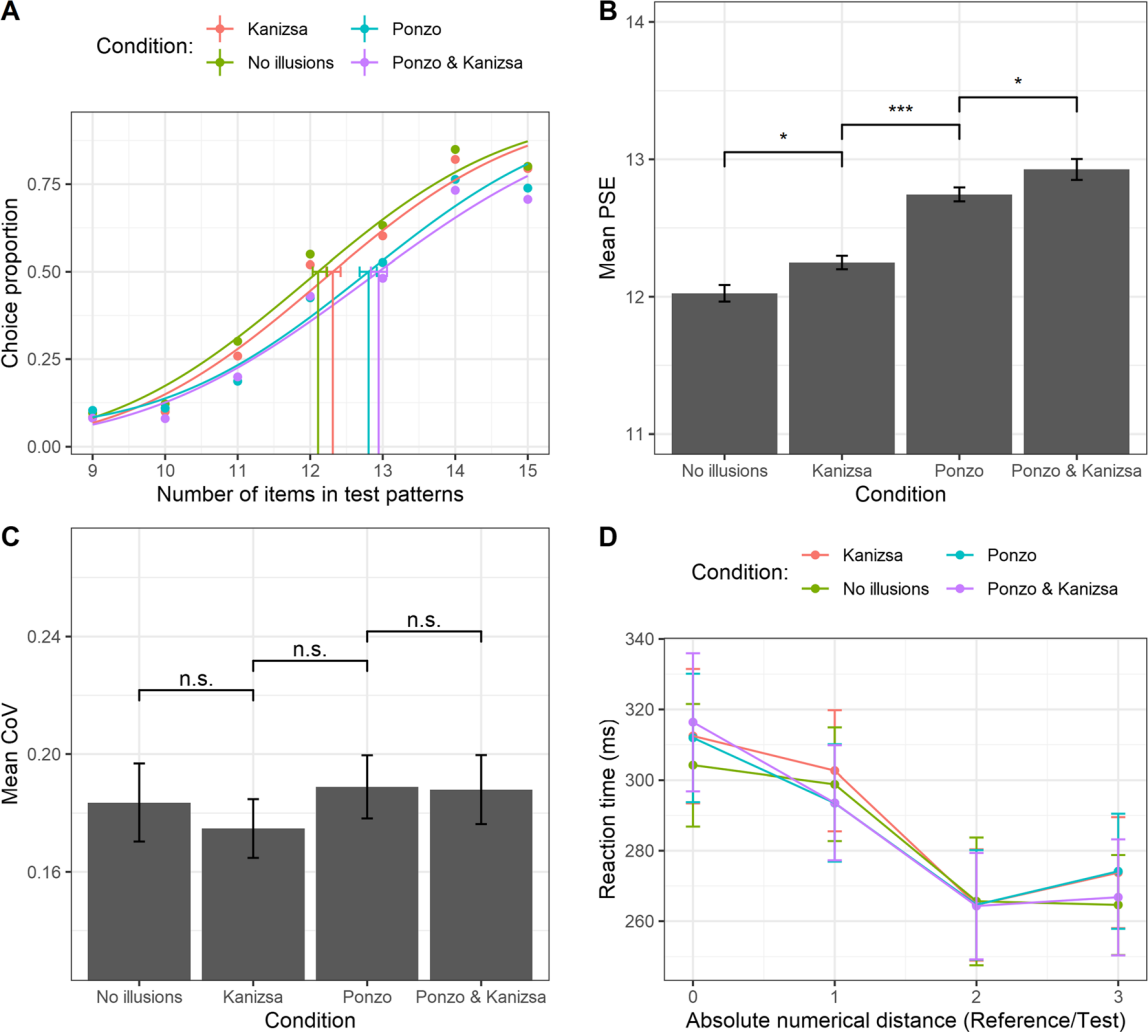
**a** Psychometric functions obtained fitting Gaussian cumulative distribution function (for each experimental condition) pooling over the aggregate data of all the subjects. Please note that this graph is reported to illustrate the statistical technique, but all subsequent analyses were done with similar functions over individual subjects. The *x*-axis represents the actual number of items in test patterns, whereas the *y*-axis shows the proportion of test patterns that were judged as more numerous than the reference. Vertical lines represent the PSE (0.5 threshold level) for each condition. The error bars represent the bootstrap 95% confidence intervals. **b** Mean PSE as a function of the experimental condition. **c** Mean CoV as a function of the experimental condition. **d** Mean RTs as a function of the experimental condition and the absolute numerical distance between reference and test stimuli. The error bars represent ±1 standard error of the mean (*SEM*). * *p* < .05; ** *p* < .01; *** *p* < .001; *ns* = nonsignificant

The analysis on the PSE (i.e., the higher the PSE the greater the underestimation bias) showed a significant effect of the experimental condition, *F*(3, 198) = 55.1, ε = .89, *p* < .001, η_p_^2^ = .45 (see Fig. 3a–b). Post hoc comparisons (Bonferroni–Holm correction) revealed a significant difference between the baseline (mean PSE ± **SD**, 12.025 ± 0.49) and the Kanizsa condition (12.24 ± 0.39), *t*(198) = −2.79, *p* = .012, the baseline and the Ponzo condition (12.74 ± 0.41), *t*(198) = −8.99, *p* < .001, the baseline and the combined condition (12.92 ± 0.62), *t*(198) = −11.26, *p* < .001, as well as between the Kanizsa condition and the Ponzo condition, *t*(198) = −6.2, *p* < .001, between the Kanizsa condition and the combined condition, *t*(198) = −8.47, *p* < .001, and crucially, between the Ponzo condition and the combined Ponzo–Kanizsa condition, *t*(198) = −2.27, *p* = .024. No significant effect of the experimental illusion condition was found over the mean CoV, *F*(3, 198) = 1.06, *p* = .36, η_p_^2^ = .016 (see Fig. 3c). To further quantify the magnitude of this null effect we also computed the Bayes factor (BF), and we found a strong evidence in favor of the null hypothesis, *BF*_*10*_ = .066. We also evaluated the relationship between individual CoVs across the illusion conditions. Results showed a significant correlation with a strong positive relationship for the acuity for each pairwise comparison (all *r* Pearson’s coefficients between .65 and .76, all *p*s < .001; see Fig. S1 and Table S1 in the Supplementary Materials)*,* suggesting that a common sensory mechanism may drive the discrimination performance across the illusion conditions.

Finally, the ANOVA on RTs showed no significant main effect of the illusion condition on RTs, *F*(3, 198) = 0.433, ε = .88, *p* = .70, η_p_^2^ = .007; *BF*_*10*_ = .004 (see Fig 3d and Table S2). A significant main effect of the absolute numerical distance between test and reference was found, *F*(3, 198) = 43.73, ε = .87, *p* < .001, η_p_^2^ = .87. Trend analysis showed a significant linear decrease of RTs when numerical distance increases, *t*(198) = −10.11, *p* < .001. Lastly, no significant interaction between variables was found, *F*(9, 594) = 0.56, ε = .73, *p* = .778, η_p_^2^ = .008. Results from Bayesian analysis suggest a strong evidence against including the interaction, *BF*_*10*_ = .0008 (e.g., full model with interaction compared with the model with only main effects; see Table S2).

## Experiment 2

In Experiment 2, participants performed the same number comparison task of Experiment 1, but in the combined condition the biases of the two illusions were modulated in a conflicting direction. That is, the Ponzo illusion triggered an overestimation bias, while the Kanizsa illusion an underestimation bias. If discrete information is processed independently from continuous features, when the two individual illusions are combined over the same stimulus in a conflicting direction, we should expect participants’ bias to be halfway as compared with the single illusory conditions, indicating that the two illusions compete against each other.

### Materials and Methods

#### Participants

A new sample of 68 participants (*M*_age_ = 30.9 years, *SD* = 12.9 years, 49 females, 55 right-handed) was recruited for this study, which was also performed online due to COVID-19 restrictions.

#### Stimuli and procedure

**Fig. 4:**
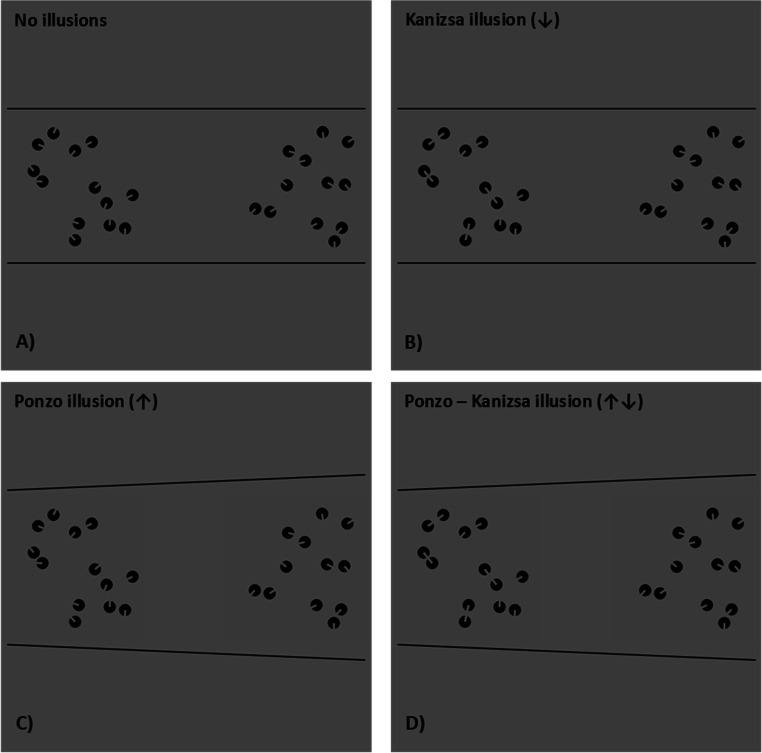
The four experimental conditions of Experiment 2. **a** The no-illusions condition, in which neither the Kanizsa nor the Ponzo illusions were presented. **b** The Kanizsa illusion condition, in which only the effect of items connectedness was manipulated, **c** The Ponzo illusion condition, in which the perceived convex-hull/density of the sets was manipulated. **d** The Ponzo–Kanizsa merged illusion, combining the effects of these last two conditions. The only difference with Experiment 1 was that the direction of the biases triggered by the Ponzo and the Kanizsa illusion were opposite, with the former that should elicit an overestimation and the latter an underestimation

The stimuli generation and the procedure were identical to that of Experiment 1. The only difference was that test stimuli were anchored to the smaller side of the Ponzo context (see Fig 4). The relative side of the reference and of the test patterns was counterbalanced and randomized across trials. The smaller side of the Ponzo illusion was randomly presented to the left or to the right, according to the test stimulus side. In this case, therefore, the directions of the biases triggered by the Ponzo and the Kanizsa illusion were opposite: while the Ponzo illusion should trigger an overestimation as compared with the reference, the Kanizsa illusion should trigger an underestimation bias.

### Results

**Fig. 5:**
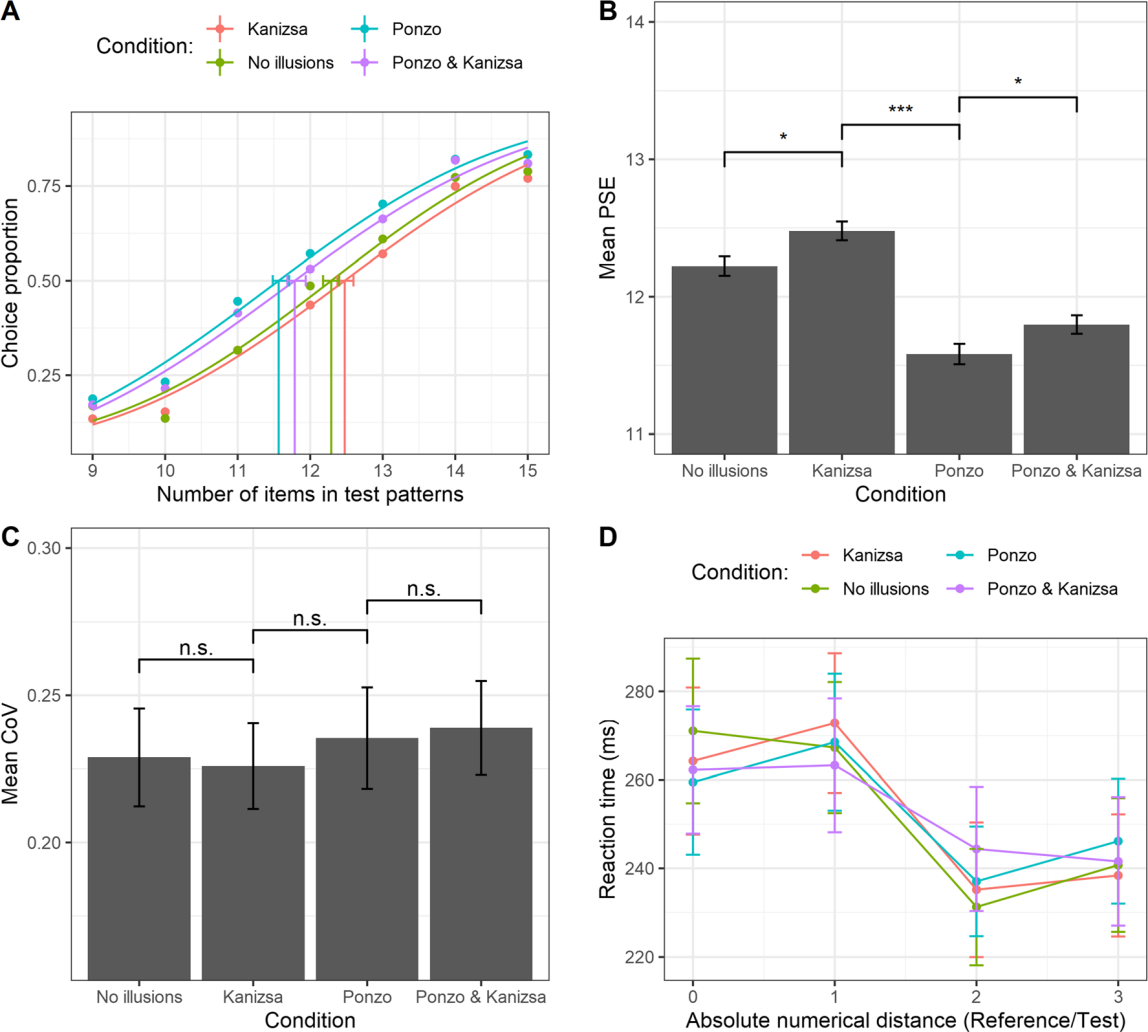
**a** Psychometric functions obtained fitting Gaussian cumulative distribution function (for each experimental condition) pooling over the aggregate data of all the subjects. Please note that this graph is reported to illustrate the statistical technique, but all subsequent analyses were done with similar functions over individual subjects. The *x*-axis represents the actual number of items in test patterns, whereas the *y*-axis shows the proportion of test patterns that were judged as more numerous than the reference. Vertical lines represent the PSE (0.5 threshold level) for each condition. The error bars represent the bootstrap 95% confidence intervals. **b** Mean PSE as a function of the experimental condition. **c** Mean CoV as a function of the experimental condition. **d** Mean RTs as a function of the experimental condition and the absolute numerical distance between reference and test stimuli. The error bars represent ±1 standard error of the mean (*SEM*). * *p* < .05; ** *p* < .01; *** *p* < .001; *ns* = nonsignificant

Data were analyzed analogously to Experiment 1. The ANOVA on PSE showed a significant effect of the experimental condition, *F*(3, 201) = 36.3, *p* < .001, η_p_^2^ = .35 (see Fig 5a–b). Post hoc comparisons (Bonferroni–Holm correction) revealed a significant difference between the baseline (12.22 ± 0.58) and the Kanizsa condition (12.47 ± 0.56), *t*(201) = −2.69, *p* = .015, the baseline and the Ponzo condition (11.58 ± 0.61), *t*(201) = 6.74, *p* < .001, the baseline and the combined Ponzo–Kanizsa condition, (11.79 ± 0.55), *t*(201) = −4.47, *p* < .001, between the Kanizsa condition and the Ponzo condition, *t*(201) = 9.43, *p* < .001, the Kanizsa condition and the combined condition, *t*(201) = 7.17, *p* < .001, and, crucially, between the Ponzo condition and the combined Ponzo–Kanizsa condition, *t*(201) = −2.22, *p* = .025. As expected by the direct account of numerosity, the PSE of the combined condition was between the PSE of the illusion in isolations.

No significant effect of the experimental condition was found over the mean CoV, *F*(3, 201) = 0.83, ε = .85, *p* = .46, η_p_^2^ = .012 (see Fig. 5c). Bayesian analysis suggests strong evidence in favor of the null hypothesis, *BF*_*10*_ = .049. We also evaluated the relationship between individual CoVs across the experimental conditions. As in Experiment 1, results showed a significant correlation with a strong positive relationship for the acuity for each pairwise comparison (all *r* Pearson’s coefficients between .77 and .90, all *p*s < .001, see Fig S2 and Table S3 in the Supplementary Materials)*.*

Furthermore, as in the previous experiment, no significant main effect of the experimental condition was found over RTs (7% of the data were discarded), *F*(3, 201) = 0.28, ε = .90, *p* = .81, η_p_^2^ = .004; *BF*_*10*_ = .004 (see Fig 5d and Table S4). We found a significant main effect of the absolute distance, *F*(3, 201) = 14.14, ε = .86, *p* < .001, η_p_^2^ = .17. A significant decreasing linear trend was also found over the absolute numerical distance, *t*(201) = −4.84, *p* < .001. Finally, no significant interaction between variables was found, *F*(9, 603) = 0.80, ε = .81, *p* = .58, η_p_^2^ = .012. Results from Bayesian analysis suggest a very strong evidence against including the interaction, *BF*_*10*_ = .0015 (e.g., full model with interaction compared with the model with only main effects; see Table S4).

## General discussion

In this study, across two different experiments, we document a clear role of visual segmentation mechanisms in discrete numerosity processing (e.g., Burr & Ross, 2008; Franconeri et al., 2009; Piazza et al., 2004; Verguts & Fias, 2004). Indeed, we found that IC connections in the Kanizsa condition, when manipulated in isolation, led to a numerical underestimation bias in both experiments, as more items needed to be present in the test stimulus in order to be judged as numerically equal to the reference (e.g., increase in the PSEs). These data suggest that the pair of connected objects may form a unity that is selected as input for numerosity (e.g., two dots connected by the illusory contour would be processed as one), hence triggering the change in PSE (underestimation) when the four pairs of dots were connected by ICs (see also Adriano et al., 2021). Notably, this was found despite continuous features were kept constant across baseline and Kanizsa condition, as our test stimuli had the same item spacing, the same total contour (e.g., high spatial frequency) and the same object size and convex hull (e.g., low spatial frequency), thus challenging current alternative views maintaining a key role of these variables (e.g., Allik & Tuulmets, 1991; Dakin et al., 2011; Durgin, 2008; Gebuis et al., 2016).

However, we also found a clear bias triggered by the Ponzo illusion when this illusion was presented in isolation. In line with previous studies using the Ebbinghaus illusion, we found that the Ponzo illusion led to an underestimation (Experiment 1) or an overestimation (Experiment 2) of test numerosity, depending on the context in which it was placed (see also Picon et al., 2019). These findings undoubtedly show that also the (perceived) size of the convex hull/density of the set is taken into account for decisions during the comparison task, confirming previous studies (e.g., Picon et al., 2019). Indeed, while these two illusions tap on different perceptual (i.e., the Ponzo illusion induces the perception of three-dimensional depth/distance information, while the Ebbinghaus illusion do not) and neural mechanisms (e.g., Song et al., 2011), the behavioral effects found are very similar in the context of nonsymbolic comparison tasks. All these works using size illusions (Dormal et al., 2018; Picon et al., 2019) are also in line with studies showing that visual adaptation to size (e.g., adapter stimuli were discs of different size) affects subsequent perceived numerosity. However, such a size adaptation was stronger only for numerically very large arrays (Zimmermann & Fink, 2016), compatibly with the idea that density and size may be prominent cues only for denser textured stimuli (e.g., Dakin et al., 2011), but not for sparse/lower numerosities (e.g., Anobile et al., 2017). Additionally, Anobile and colleagues (2018) recently asked participants to perform both a size and a numerosity adaptation task and found that neither discrimination thresholds nor adaptation strength correlate with each other. Crucially, our results showed that when both the Ponzo and the Kanizsa illusions were combined over the same physical stimulus, the joint effect varied according to their bias direction. More precisely, biases of each illusion summated (i.e., largest underestimation as compared with the condition in which only one illusion was presented) in Experiment 1, while they averaged and competed against each other in Experiment 2. These findings can be explained if we assume that when illusions are combined, a “discrete” information is still actually processed independently from continuous features. This provides a clear evidence against the views maintaining that perceived numerosity is simply the result of weighting a variety of continuous visual properties (Gebuis & Reynvoet, 2012b). Rather, our study indicates that both discrete elements and continuous magnitudes can simultaneously affect perceived numerosity and influence the behavior. More critically, the PSE pattern of the *combined* conditions indicates that participants integrate the bias induced by the Kanizsa-connectedness (underestimation) illusion with the bias induced by the Ponzo illusion (either overestimation or underestimation, depending on the experimental manipulation). Indeed, if participants actually ignored discrete numerosity, no difference should be found between the *combined* condition and the Ponzo condition in isolation, since in both conditions we have exactly the same continuous cues (as well as between the no-illusions condition and the Kanizsa condition). This strongly suggests that in the merged condition there is a *combined* effect of both information—namely, of discrete (manipulated by ICs) and continuous information (manipulated by the Ponzo illusion). Notably, neither of the two types of information dominated the other, and hence no factor was lost or ignored—they were simply combined (according to the direction of biases in each experiment).

Yet, contrary to the holistic view, according to which numerosity is processed along with other perceptual variables to construct a sense of magnitude (Leibovich et al., 2017), our data suggest that both discrete elements and continuous variables would be independently integrated together to guide the behavior. Evidence for this integrative process comes from the pattern of results that we observed here over the PSEs across experimental illusion conditions, suggesting that two independent magnitude information might be at play. The fact that, as expected by Weber’s law, CoV (e.g., numerical acuity) and RTs were constant across illusion conditions, strongly rules out task difficulty as a possible confounding variable accounting for our findings (e.g., change in PSE across conditions), rather suggesting a genuine equal perceptual discriminability of stimuli across illusion contexts. However, the strong correlations across experimental conditions of individual precision (indexed by the CoV) and the fact that CoV was stable across illusion conditions also suggest that a common sensory mechanism may operate and drive the discrimination performance in the numerical task, following Weber’s law. The independence between numerosity and size information is also indirectly supported by studies showing that both continuous physical dimensions (e.g., item size or cumulative surface) and discrete number information may be automatically extracted in Stroop-like tasks even when they are irrelevant to the task (e.g., Hurewitz et al., 2006; Nys & Content, 2012) or when dot arrays are passively viewed (Van Rinsveld et al., 2020), and they may interact or compete for behavioral control perhaps in a late decisional stage (Franconeri et al., 2009; Leibovich & Henik, 2014). Furthermore, it has been shown that discrimination threshold for numerosity, but not for size judgement, is impaired in dyscalculic subjects (Anobile, Cicchini, et al., 2018), with other studies reporting that arithmetical education selectively improves acuity in nonsymbolic numerical discrimination, but not in size discrimination (Piazza et al., 2013). These studies more broadly challenge the idea that continuous cues could be at the core of the development of mathematical abilities.

In conclusion, this study challenges recent theoretical accounts according to which people would not extract numerosity independently from other continuous magnitudes (Gebuis & Reynvoet 2012a, 2012b; Gebuis et al., 2016). Our study, indeed, testifies the existence of a distinct sense of number that allows perceiving discrete numerosities information exploiting segmentation and perceptual organization, but integrating also other features of the visual input, including continuous magnitudes information such as size. That is, we demonstrate that subjective numerosity could be the result of a flexible combination between continuous and discrete information from the visual scene. These findings indirectly support the hypothesis of a general mechanism that allows for processing of both discrete (i.e., number) and continuous dimensions (i.e., space) in parietal areas (e.g., Walsh, 2003), and points to the need of more comprehensive theoretical views that should account for the operations by which both discrete elements and continuous variables signals are computed and integrated together as relevant cues for extracting numerosity information from the visual stream (e.g., Cantrell & Smith, 2013).

## Supplementary Information

ESM 1 (CSV 9 kb)

ESM 2 (CSV 5 kb)

ESM 3 (CSV 8 kb)

ESM 4 (CSV 5 kb)

ESM 5 (DOCX 497 kb)

## Data Availability

The data sets generated during the current study are available online as Supplementary Materials.
