# Supplementary material for: Number is not just an illusion: Discrete numerosity is encoded independently from perceived size
Source: Psychon Bull Rev. 2021 Aug 11;29(1):123–33. doi: 10.3758/s13423-021-01979-w (PMC8356546; doi:10.3758/s13423-021-01979-w)
Supplement: Supplementary file 5 — (DOCX 497 kb) [file 13423_2021_1979_MOESM5_ESM.docx]

**Supplementary Materials**

**Number is not just an illusion:**

**Discrete numerosity is encoded independently from perceived size**

Andrea Adriano^1*^, Luisa Girelli^1,2#^, & Luca Rinaldi^3,4#^

*^1^ Department of Psychology, University of Milano-Bicocca (Italy)*

*^2^ NeuroMI, Milan Center for Neuroscience, Milano (Italy)*

*^3^ Department of Brain and Behavioral Sciences, University of Pavia, Pavia (Italy)*

*^4^ Cognitive Psychology Unit, IRCCS Mondino Foundation, Pavia (Italy)*

**^*^** Corresponding author: A. Adriano. Dipartimento di Psicologia, Università degli Studi di Milano-Bicocca, Piazza dell’Ateneo Nuovo 1, Edificio U6, 20126 Milano, Italy.

E-mail: [a.adriano1@campus.unimib.it](mailto:a.adriano1@campus.unimib.it)

^#^ These authors contributed equally to this work.

Experiment 1


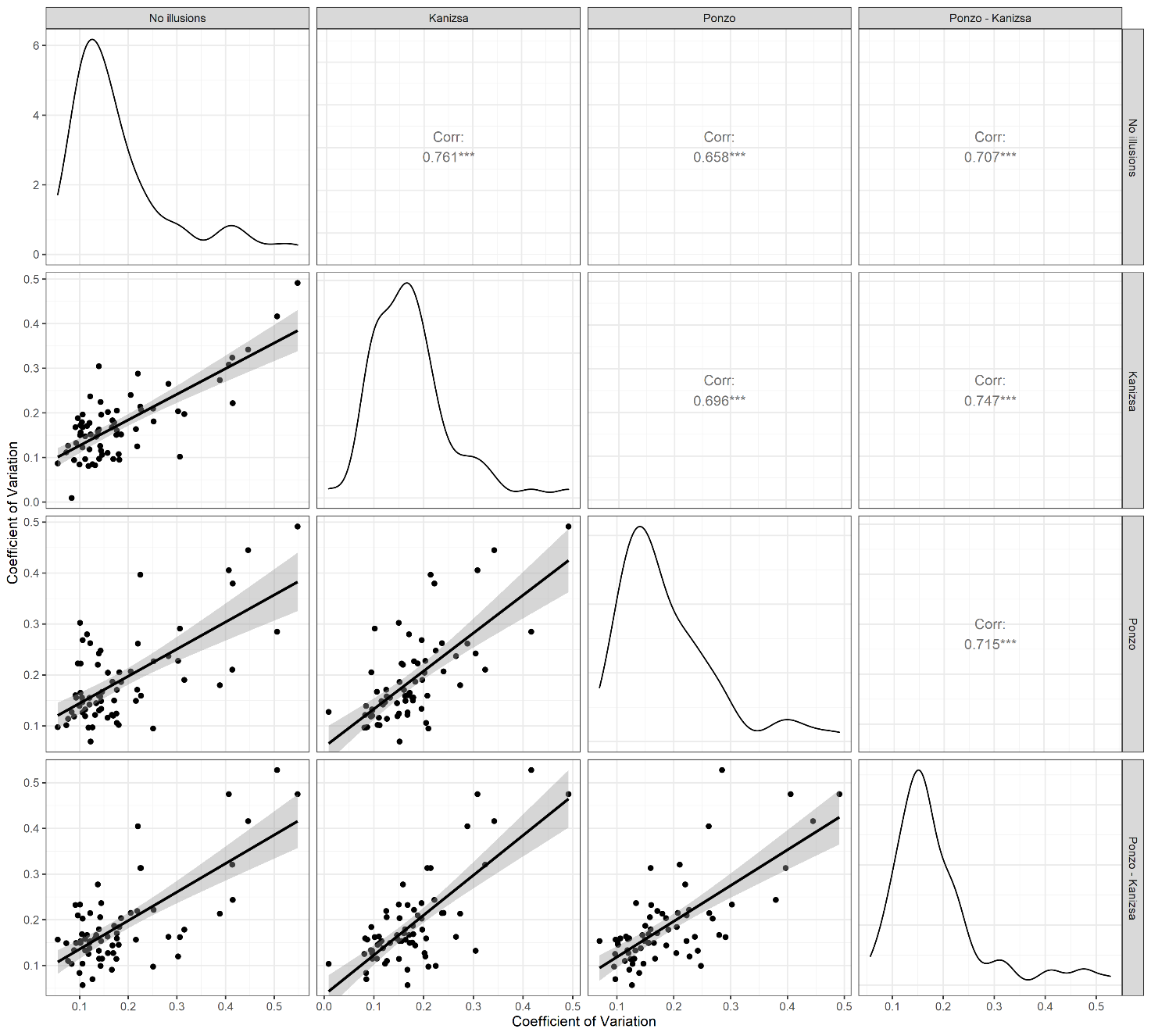


**Figure S1:** Scatterplots showing the correlations among conditions. Shaded regions represent the 95% CI of the correlation line.

|  | | | | | | | | | | | |
| --- | --- | --- | --- | --- | --- | --- | --- | --- | --- | --- | --- |
| **Variable** | |  | | **Kanizsa** | | **Ponzo** | | **No illusions** | | **Ponzo & Kanizsa** | |
| 1. Kanizsa |  | n |  | — |  |  |  |  |  |  |  |
|  |  | Pearson's r |  | — |  |  |  |  |  |  |  |
|  |  | p-value |  | — |  |  |  |  |  |  |  |
| 2. Ponzo |  | n |  | 67 |  | — |  |  |  |  |  |
|  |  | Pearson's r |  | 0.696 | *** | — |  |  |  |  |  |
|  |  | p-value |  | < .001 |  | — |  |  |  |  |  |
| 3. No illusions |  | n |  | 67 |  | 67 |  | — |  |  |  |
|  |  | Pearson's r |  | 0.761 | *** | 0.658 | *** | — |  |  |  |
|  |  | p-value |  | < .001 |  | < .001 |  | — |  |  |  |
| 4. Ponzo & Kanizsa |  | n |  | 67 |  | 67 |  | 67 |  | — |  |
|  |  | Pearson's r |  | 0.747 | *** | 0.715 | *** | 0.707 | *** | — |  |
|  |  | p-value |  | < .001 |  | < .001 |  | < .001 |  | — |  |
|  | | | | | | | | | | | |
| * *p* < .05, ** *p* < .01, *** *p* < .001 | | | | | | | | | | | |

**Table S1:** CoV correlation values among conditions.

| **Model Comparison** | | | | | | | | | | | |
| --- | --- | --- | --- | --- | --- | --- | --- | --- | --- | --- | --- |
| **Models** | | **P(M)** | | **P(M\|data)** | | **BF _M_** | | **BF _10_** | | **error %** | |
| Null model (incl. subject) |  | 0.200 |  | 4.701e -31 |  | 1.881e -30 |  | 1.000 |  |  |  |
| Distance |  | 0.200 |  | 0.995 |  | 843.858 |  | 2.117e +30 |  | 0.672 |  |
| Distance + Condition |  | 0.200 |  | 0.005 |  | 0.019 |  | 1.003e +28 |  | 1.778 |  |
| Distance + Condition + Distance  ✻  Condition |  | 0.200 |  | 4.015e  -6 |  | 1.606e  -5 |  | 8.541e +24 |  | 1.864 |  |
| Condition |  | 0.200 |  | 2.032e -33 |  | 8.128e -33 |  | 0.004 |  | 0.595 |  |
|  | | | | | | | | | | | |
| *Note.*  All models include subject | | | | | | | | | | | |

**Table S2:** Bayesian ANOVA on the CoV.

Experiment 2


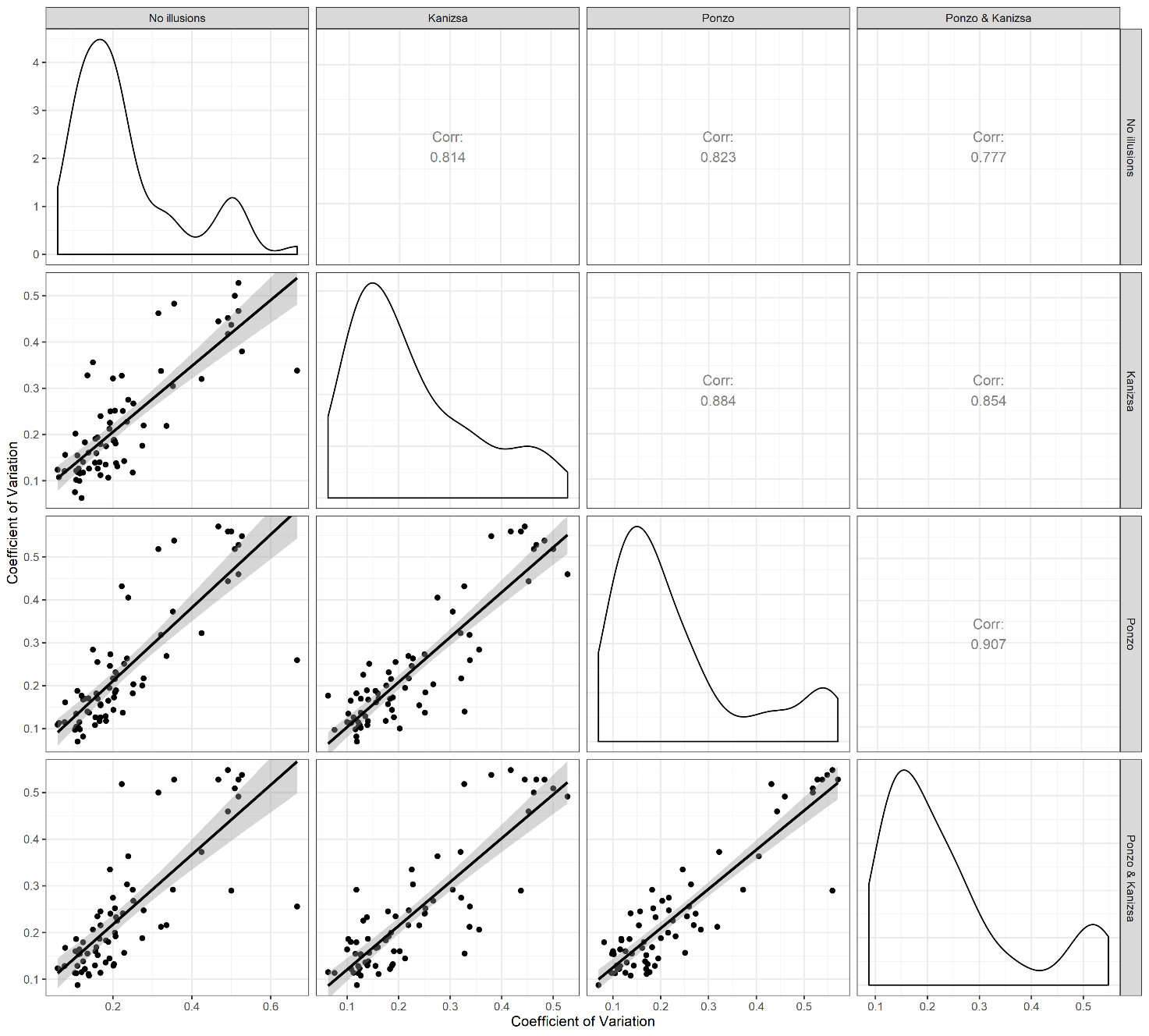


**Figure S2:** Scatterplots showing the correlations among conditions. Shaded regions represent the 95% CI of the correlation line.

|  | | | | | | | | | | | |
| --- | --- | --- | --- | --- | --- | --- | --- | --- | --- | --- | --- |
| **Variable** | |  | | **Kanizsa** | | **Ponzo** | | **No illusions** | | **Ponzo & Kanizsa** | |
| 1. Kanizsa |  | n |  | — |  |  |  |  |  |  |  |
|  |  | Pearson's r |  | — |  |  |  |  |  |  |  |
|  |  | p-value |  | — |  |  |  |  |  |  |  |
| 2. Ponzo |  | n |  | 68 |  | — |  |  |  |  |  |
|  |  | Pearson's r |  | 0.884 | *** | — |  |  |  |  |  |
|  |  | p-value |  | < .001 |  | — |  |  |  |  |  |
| 3. No illusions |  | n |  | 68 |  | 68 |  | — |  |  |  |
|  |  | Pearson's r |  | 0.814 | *** | 0.823 | *** | — |  |  |  |
|  |  | p-value |  | < .001 |  | < .001 |  | — |  |  |  |
| 4. Ponzo & Kanizsa |  | n |  | 68 |  | 68 |  | 68 |  | — |  |
|  |  | Pearson's r |  | 0.854 | *** | 0.907 | *** | 0.777 | *** | — |  |
|  |  | p-value |  | < .001 |  | < .001 |  | < .001 |  | — |  |
|  | | | | | | | | | | | |
| * *p* < .05, ** *p* < .01, *** *p* < .001 | | | | | | | | | | | |

**Table S3:** CoV correlation values among conditions.

| **Model Comparison** | | | | | | | | | | | |
| --- | --- | --- | --- | --- | --- | --- | --- | --- | --- | --- | --- |
| **Models** | | **P(M)** | | **P(M\|data)** | | **BF _M_** | | **BF _10_** | | **error %** | |
| Null model (incl. subject) |  | 0.200 |  | 1.479e -10 |  | 5.916e -10 |  | 1.000 |  |  |  |
| Distance |  | 0.200 |  | 0.996 |  | 1030.224 |  | 6.735e +9 |  | 0.654 |  |
| Distance + Condition |  | 0.200 |  | 0.004 |  | 0.016 |  | 2.611e +7 |  | 1.238 |  |
| Distance + Condition + Distance  ✻   Condition |  | 0.200 |  | 5.826e  -6 |  | 2.331e  -5 |  | 39391.509 |  | 1.148 |  |
| Condition |  | 0.200 |  | 5.443e -13 |  | 2.177e -12 |  | 0.004 |  | 0.575 |  |
|  | | | | | | | | | | | |
| *Note.*  All models include subject | | | | | | | | | | | |

**Table S4:** Bayesian ANOVA on the CoV.
